# Roux-en-Y gastric bypass surgery induced oxalosis and acute kidney injury: A case report

**DOI:** 10.1016/j.amsu.2021.103088

**Published:** 2021-11-20

**Authors:** Zahra Askari, Javad Boskabadi, Saeed Kargar-Soleimanabad, Farhad Gholami

**Affiliations:** a Department of Toxicology and Pharmacology, Faculty of Pharmacy, Mazandaran University of Medical Sciences, Sari, Iran; b Department of Clinical Pharmacy, Faculty of Pharmacy, Mazandaran University of Medical Sciences, Sari, Iran; c Student Research Committee, Faculty of Medicine, Mazandaran University of Medical Sciences, Sari, Iran; d Department of Internal Medicine, Faculty of Medicine, Mazandaran University of Medical Sciences, Sari, Iran

**Keywords:** Acute kidney injury (AKI), Gastric bypass surgery, Secondary oxalosiss, CKD, Case report

## Abstract

**Introduction and importance:**

Diabetes mellitus and hypertension are two conditions that can coexist in obese individuals. Roux-en-Y gastric bypass (RYGB) surgery, are used to control obesity. Complications such as steatorrhea, hyperoxaluria, and decreased bone mineral density, may occur after RYGB.

**Case presentation:**

A 58-year-old woman referred to the emergency department complaining of pain on the right side of her lower abdomen. Her past medical history was RYGB surgery, COVID-19 with 40% pulmonary involvement, and Chronic Kidney Disease (CKD). Rapid progressive glomerulonephritis (RPGN) was predicted based on extensive laboratory test results. A kidney biopsy demonstrated oxalate nephropathy. Along with the findings from the kidney biopsy, acute tubulointerstitial nephritis with tubular injury secondary oxalosis was diagnosed.

**Clinical discussion:**

RYGB surgery and chronic kidney disease, can increase the risk of secondary oxalosis. Recent studies introduce enteric hyperoxaluria as an important marker for diagnosing end-stage kidney disease. Renal biopsy is often prescribed for absolute recognition of oxalosis. On the other hand, our patient has a recent history of COVID-19 infection. The use of anti-Covid-19 drugs in patients with renal insufficiency should be considered with caution.

**Conclusion:**

It is important to monitor kidney function following RYGB surgery, particularly in patients with underlying diseases such as diabetes or hypertension.

## Highlights

• Diabetes mellitus and hypertension are two conditions that can coexist in obese individuals.
• Roux-en-Y gastric bypass (gastric bypass), are used to control obesity.
• Gastric bypass surgery and chronic kidney disease both increase the risk of secondary oxalosis.

## 1 Introduction

Diabetes mellitus and hypertension are two conditions that can coexist in obese individuals. On the other hand, obesity independent of diabetes or hypertension can cause increase glomerular filtration and glomerular capillary pressure related to early stages of chronic kidney disease [1].

Different strategies, including bariatric surgery, sleeve gastrectomy, and Roux-en-Y gastric bypass (gastric bypass), are used to control obesity. Roux-en-Y gastric bypass (RYGB) surgery leads to long-term weight loss and reduced incidence and remission of diabetes and hypertension. However, this process is associated with various metabolic and mineral disorders [2]. It was demonstrated that steatorrhea, hyperoxaluria, and decreased bone mineral density are seen after RYGB [3].Oxalates have a significant physiological effect on the kidneys. While minute calcium oxalate crystals are excreted in the urine, large oxalate crystal compounds obstruct the kidney tubules, and sedimenting calcium oxalate in kidney tissue results in acute tubular injury [4].

It was reported that, in 1–3.5 years of follow-up after RYGB, the incidence of hyperoxaluria was 42–67% [5]. Oxalosis occurs by four different reasons. First with increasing to use oxalate or substances that metabolize to oxalate such as ascorbic acid (vitamin C), oxalate-rich foods (nuts, rhubarb, beets, sesame seeds) and ingestion of ethylene glycol [[6], [7], [8]]. Second with stimulating oxalate absorption in surgeries and intestinal diseases such as RYGB surgery and inflammatory bowel disease (IBD) respectively. Kidney stones frequently reported in IBD and RYGB because in inflammatory conditions, absorption is impaired and not performed properly. Hypocitraturia and hypomagnesiuria and other impact of these metabolic disturbances are the main factors which could explain appearance of kidney stones mainly of oxalate of calcium [7,[9], [10], [11], [12]].

Third, decreased oxalate excretion in Chronic Kidney Disease (CKD). CKD gradually impairs kidney function over months to years. Diabetes mellitus (DM) and high blood pressure are important risk factors for CKD [13].

The fourth reason is a deficiency of a specific vitamin, such as thiamine (B1) or pyridoxine (B6). A recent study demonstrated that vitamin B6 deficiency significantly enhances the activities of hepatic glycolate oxidase and glycolate dehydrogenase, whereas vitamin B1 deficiency only amplifies the activity of the glycolate oxidase enzyme. Consequently, thiamine and pyridoxine deficiencies stimulate oxalate formation [[14], [15], [16]].

This case report discussed acute kidney injury caused by secondary oxalosis following Roux-en-Y Gastric Bypass Surgery in conjunction with Chronic Kidney Disease (CKD).

## 2 Case presentation

### 2.1 Clinical history and initial laboratory data

This study was conducted according to the Declaration of Helsinki principles, as well as CARE guidelines and methodology [17]. A 58-year-old woman with right abdominal pain was referred to the emergency department of Imam Khomeini hospital located in Sari, North of Iran. Her family medical history was negative and her past medical history included gastric bypass surgery approximately one year ago, COVID-19 (approximately seven months ago) with 40% pulmonary involvement, chronic kidney disease (CKD), hypertension, diabetes mellitus (FBS = 339mg/dl), kidney stones, and hypothyroidism.

Seven months ago, her CKD was diagnosed at stage 3 with a serum creatinine level of 2mg/dl and an estimated glomerular filtration rate (GFR) of 33mL/min/1.73 m^2^. Her Medications included pregabalin 50mg/hs, lamotrigine 50mg/hs haloperidol 0.5mg/hss, quetiapine 25mg/1/2hs, levothyroxine 0.1mg/d, duloxetine10mg/d, amlodipine5mg/d, atorvastatin 20/d, vitamin B1 300mg/d, and detemir insulin 20 IU/d. Additionally, she received a remdesivir for less than ten days during the COVID-19 infection.

Initial laboratory data are summarized in Table 1. Serum creatinine and serum urea nitrogen levels were 9.9 mg/dL and 220mg/dL, respectively, at emergency department. Urine analysis revealed no evidence of cast or crystal. A kidney ultrasound revealed a cortical cyst (30mm) in the right kidney's lower bridge, as well as a slightly increased parenchymal echo. Another ultrasound examination revealed no evidence of hydronephrosis, calcifications, or kidney stones. On the other hand, in first day of hospitalization, proteinuria with a 373mg/24h protein excretion was reported.

**Table 1:** Initial laboratory data.

Test	Result	Reference range
**K**	3.2meq/l	3.5–5.5
**Na**	136meq/l	135–145
**Hgb**	9.4g/dl	11.7–16
**WBC**	11.6 × 10*3	4.5–11
**HCT**	27.4%	35–47
**CREATININE**	9.9mg/dl	0.6–1.3
**Urea**	220mg/dl	13–43
**AST**	112U/L	<35
**ALT**	149U/L	<35
**Alb**	4.3	3.5–5.2
**PT**	12Sec	11–13
**PTT**	31Sec	24–35
**INR**	1	1–1.5
pH**(ARTERIAL BLOOD)**	7.18	7.35–7.45
**Hco3**	8.0mmol/L	23–26
**C3**	82mg/dl	90–180
**C4**	23mg/dl	10–40
**FBS**	339mg/dl	70–115
**CRP**	5.4mg/dl	Up to 6
**Uric acid**	8.2mg/dl	2.6–8.6
**Phosphorus**	7.7mg/dl	2.5–4.8
**Calcium**	7.8 mg/dl	8.5–10.5
**Ck-MB**	19U/L	<24
**PTH**	398pg/ml	11–67
**ESR**	39mm/hour	0–20
**Urine protein 24h**	373mg/24h	24–141
**Urine creatine24h**	0.5g/24h	0.6–1.2
**Urine volum24h**	1500ml/24h	600–1800
**urea(fourth day of hospitalization)**	116mg/dl	13–43
**Creatinine (fourth day of hospitalization)**	5.6mg/dl	0.6–1.3
**Urine-Alb**	185 mg/24h	<30 mg/24 h

### 2.2 Kidney biopsy

A biopsy of the kidney was performed. The microscopic examination revealed glomerular enlargement with mild mesangial expansion and hypercellularity in both arterioles without the formation of nodules or hyalinosis, consistent with diabetic nephropathy. Observation by light microscopy showed interstitial infiltration of lymphocytes with tubulitis that revealed interstitial nephritis and. numerous calcium oxalate crystals accumulated within tubules, resulting in tubular injury.Immunofluorescence microscopy was used to stain frozen sections containing two glomeruli with IgG, IgA, IgM, C1q, C4, C3, Fibrinogen, and polyvalent antisera that demonstrated negative immune reaction.

### 2.3 Diagnosis

Specialists predicted rapid progressive glomerulonephritis (RPGN) based on significant laboratory test results (Table 1, Table 2). As well as the findings from the kidney biopsy, acute tubulointerstitial nephritis with tubular injury from secondary oxalosis was diagnosed.

**Table 2:** Follow up laboratory Data.

Test	Result	Reference range
**K**	4.77meq/l	3.5–5.5
**Na**	140meq/l	135–145
**CREATININE**	6.19mg/dl	0.6–1.3
**Urea**	111mg/dl	13–43
**AST**	27U/L	<35
**ALT**	49U/L	<35
**PT**	14.1Sec	11–13
**Activated PTT**	31.3Sec	24–35
**INR**	1	1–1.5
pH**(ARTERIAL BLOOD)**	6.1	7.35–7.45
**Glucose(Fasting)**	192mg/dl	74–100
**Hb.A1c**	8.4%hb	4.8–5.9
**C3**	63.5mg/dl	90–180
**C4**	11.7mg/dl	10–40
**24hrs.urine protein**	0.19gr/24hrs	0–0.15
**24hrs.urine oxalate**	59mg/24hrs	Up to55
**Phosphorus**	6.1mg/dl	2.5–4.8
**Calcium**	7.5 mg/dl	8.5–10.5
**Covid 19(sars-cov-2)IgM**	Negative	Negative:<0.9
**Covid 19(sars-cov-2)IgG**	Negative	Negative:<0.9
**HBs/Ag(CLIA)**	Non-reactive	
**HCV/Ab(CLIA)**	Non-reactive	
**PTH**	815pg/ml	11–67
**Ferritin**	179.4ng/ml	Female>40y:10-260
**25(OH)Vit.D Total**	16.1ng/ml	10–30

### 2.4 Clinical treatment and follow-up

During the hospitalization period, emergency hemodialysis was performed, and the patient was treated with methylprednisolone 500mg (only the first three days) and then exchanged to prednisolone 75mg/d. Additionally, potassium citrate (UROCITRA ®) 10mEq/BD, sevelamer (Renagel ®) 800mg TDS, hydrochlorothiazide 50mg/BD, vitamin B6 10mg/BD, and hydration were prescribed following an emergency report of biopsy results. After the fourth day of hospitalization, serum creatinine and serum urea nitrogen levels decreased to 5.6mg/dL and 116mg/dL, respectively (Table 1), and kidney function was slightly improved through hemodialysis therapy and medication. The patient was discharged from the hospital on a long-term treatment schedule of twice-weekly hemodialysis.

## 3 Discussion

We reported the patient with AKI on CKD and hyperoxaluria. In addition to CKD, her past medical history, included diabetes mellitus, gastric bypass surgery, COVID-19, hypertension, and hypothyroidism. Roux-en-Y gastric bypass surgery and possibility of inflammation can injure the gastrointestinal tract and cause oxalate nephropathy. Both passive diffusion and active transport can perform oxalate absorption. Oxalate crystals can cause tubular damage, interstitial fibrosis and progression of kidney disease.Fatty acids and bile salts stimulate passively oxalate fluxes [18].

Since weight loss following bariatric surgery can affect both creatinine generation and serum creatinine levels, it has been demonstrated that bariatric surgery can cause CKD. Because serum creatinine is dependent on muscle mass in the body, it is not a sensitive indicator. Following RYGB, muscle mass decreased in order to facilitate weight loss. Recognize the extent of renal damage and renal clearance is critical [7,19,20].

Hypocalcemia, Hyperparathyroidism, and Hyperphosphatemia were observed in the patient. Calcium is typically bound to oxalate in the intestine, but following bypass surgery, calcium binds to fatty acids and a decrease in free intestinal calcium results in an increase in unbound and free oxalates. After absorption in the large bowel, oxalate is transferred to the kidney and dissolves in the kidney tissue. A low-oxalate diet and sufficient calcium can control secondary oxalosis because calcium acts as an oxalate chelator [10,[21], [22], [23]].

Forward to the study results, serum C3 and urinary C3 measurement can be an accurate marker for glomerulonephritis because it is indicative of C3 deposition in glomerular capillary walls. Urinary C3 excretion indicated the importance of adequate countermeasures for disease control. Both low serum C3 levels and elevated urinary C3 levels may be sensitive indicators of glomerular disease and nephrotic syndrome [24].

Recent studies introduce enteric hyperoxaluria as an important marker for diagnosing end-stage kidney disease. Renal biopsy is often prescribed for absolute recognition of oxalosis. Specific transporters have an important role in oxalate absorption or secretion, and these factors require further studies [14,25].

Approximately seven months before the study, the patient used remdesivir for a brief time for COVID-19 treatment. Remdesivir may cause a few complications in the kidneys and liver during medical treatment for COVID-19 infection. Remdesivir is counter-indicated in patients with liver disease and dialysis patients or those with transient AKI. Therefore, in these patients, prescribing remdesivir requires adequate caution [26].

## 4 Conclusion

This report presented an acute kidney injury caused by secondary oxalosis following gastric bypass surgery in conjunction with chronic kidney disease. Oxalosis and mild mesangial expansion were diagnosed via kidney biopsies. This recognition is critical for specialists and surgeons to keep in mind how critical it is to monitor kidney function following gastric bypass surgery, particularly in patients with underlying diseases such as diabetes or hypertension, or in patients treated with remdesivir (which excretes approximately 18%t of its dose in the urine) during COVID-19 infection.

## Ethics approval

The study was approved by our local ethics committee.

## Funding sources

This research received no funding.

## Informed consent

Written informed consent was obtained from the patient for publication of this case report and accompanying images. A copy of the written consent is available for review by the Editor-in-Chief of this journal on request.

## Authorships

F.GH involved in interpretation and collecting of data, and editing the manuscript. JB, ZA and S.K involved in writing, editing and preparing the final version of manuscript. All authors reviewed the paper and approved the final version of the manuscript**.**

## Registration of research studies

1. Name of the registry:
2. Unique Identifying number or registration ID:
3. Hyperlink to your specific registration (must be publicly accessible and will be checked):

## Data availability statement

The data are available with the correspondence author and can be achieved on request.

## Guarantor

Dr, Farhad Gholami.

## Provenance and peer review

Not commissioned, externally peer-reviewed.

## Declaration of competing interest

The authors confirm that this article content has no conflict of interest.
